# Supplementary material for: Provenancing 16th and 17th century CE building timbers in Denmark–combining dendroprovenance and Sr isotopic analysis
Source: PLoS One. 2023 Feb 9;18(2):e0278513. doi: 10.1371/journal.pone.0278513 (PMC9910641; doi:10.1371/journal.pone.0278513)
Supplement: S1 File — Daly, A., 2016. Dendrokronologisk undersøgelse af tømmer fra Algade 61 Tiendeladen 7, Aalborg, NJM 6465. dendro.dk report 2016:11, Copenhagen. (PDF) [file pone.0278513.s001.pdf]

Dendrokronologisk undersøgelse af tømmer fra Algade 61 Tiendeladen 7, Aalborg, NJM 6465

af
Aoife Daly, ph.d.
Dendro.dk rapport 11 : 2016
Indsendt af Christian Klinge, Nordjyllands Historiske Museum.

I denne rapport beskrives de dendrokronologiske analyser af prøver fra tømmer fra et stående hjørnehus på adressen Algade 61 i Aalborg. Prøver fra fem tømmerstykker er indsendt, og alle er af *Quercus sp*., eg.

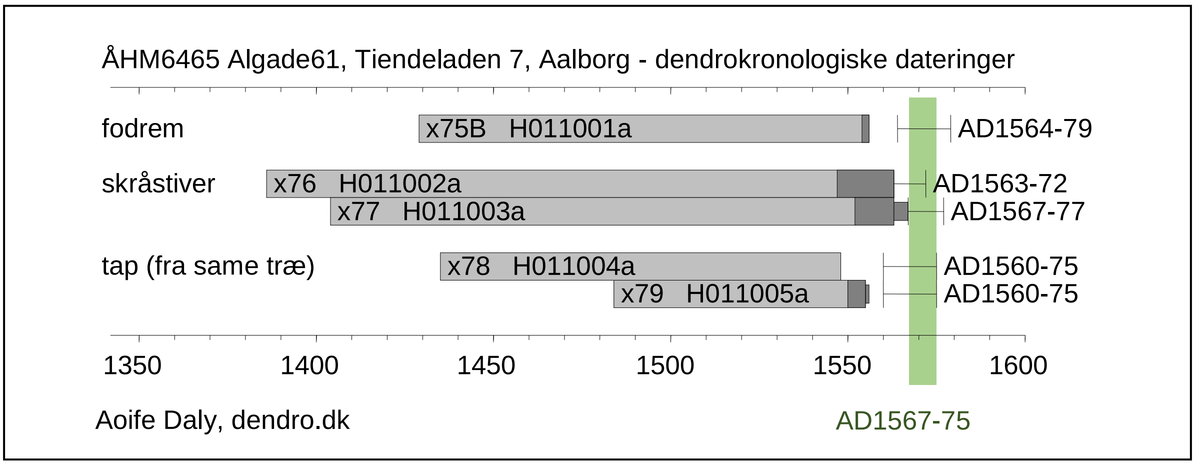

Fig. 1. Algade 61 Tiendeladen 7, Aalborg. Dateringsdiagram.

Alle fem prøver er dateret. Undersøgelsen viser, at to af prøverne, x78 og x79, kan komme fra samme træ, da de udviser meget ensartede vækstmønstre. Disse to er sammenregnet til én årringskurve H011ST4&5, som repræsenterer træet.

			H011001a	H011002a	H011003a	H011004a	H011005a
Average H011M001		H011001a	*	3,04	3,39	3,59	2,71
		H011002a	3,04	*	5,67	2,29	2,18
		H011003a	3,39	5,67	*	3,09	3,3
	Same tree H011 ST4&5	H011004a	3,59	2,29	3,09	*	10,97
		H011005a	2,71	2,18	3,3	10,97	*

Tabel 1. Algade 61 Tiendeladen 7, Aalborg. Resultatet af synkroniseringerne prøverne imellem.

Fire af de fem undersøgte prøver har splintved bevaret. Ingen har dog barkkant. Den yngste målbare årring, som blev dannet i 1563 e.Kr., er på prøve x77. Prøven har yderligere 4 årringe på ydersiden som ikke kunne måles, da de var for nedbrudte. Ved tillæg for manglende splintved er fældningstidspunktet for træet, som prøven kommer fra, beregnet til ca. 1567-77 e.Kr.
Det beregnede fældningstidspunkt for prøverne x78 og x79 falder noget tidligere, ca. 1560-75 e.Kr. Hvis vi kan antage, at træerne til bygningen blev fældet samtidigt, kan dette have fundet sted ca. 1567-75 e.Kr., markeret med grønt i fig. 1.

Filenames	-	-	H011M001	
-	start	dates	AD1386	
-	dates	end	AD1563	
Swedish chronologies				
SM000012	AD1125	AD1720	6,36	Sverige Vest (Bråthen 1982)
SM100001	AD1310	AD1539	6,22	Ystad område (Bartholin pers comm)
East Danish chronologies (can contain imports)				
B0275Morange2	AD1331	AD1557	9,56	Copenhagen Gammel Strand 24 timber IMPORTS (Daly unpubl)
B028M003	AD1386	AD1576	7,64	Køge Havn 4 timbers (Daly 2013b)
2121M002	AD1052	AD1596	7,31	Suså Næstved all posts (Daly 2001b)
B027Mpurple2	AD1275	AD1532	6,95	Copenhagen Gammel Strand 12 timbers IMPORTS (Daly unpubl)
West Danish chronologies (some contain imports)				
H009M001	AD1410	AD1613	8,81	Strandgade Nibe bolværk A6 3 timbers (Daly 2016)
Z040M001	AD1386	AD1567	7,64	Gåsehage Randers 2 timbers (Daly 2009)
8127M001	AD846	AD1771	7,14	Ålborg Østerå + Boulevarden 67 timbers (Daly 2000a, 2001a)
4077M00Z re...	AD1310	AD1546	6,81	Nyborg slot Renaissance phase 38 timbers (Daly 1999, 2007)
6094m002	AD1450	AD1660	6,50	Funder kirke 4 timbers (Daly 2002)
West Swedish TRADED timber				
q415029m04	AD1356	AD1540	7,83	Evangelistas altarpiece Seville Cathedral 29 planks (Domínguez pers comm)
Ep3mnall	AD1361	AD1539	7,75	Stirling Castle Scotland episode 3 IMPORTS (Crone pers comm)
EP41592	AD1390	AD1592	7,56	Stirling Castle Scotland episode 4 IMPORTS (Crone pers comm)
Shipwrecks & barrels				
Z073m001	AD1385	AD1574	9,15	Barcode ship 14 Oslo 3 timbers (Daly 2011)
Z141M001	AD1394	AD1529	7,82	Klippan 2 shipwreck Västergötland 11 timbers (Daly 2015b)
00652M02	AD1405	AD1607	6,89	B&W-Grunden Copenhagen vrag 2, 2 trees (Daly 2000b)
Z119M001	AD1317	AD1573	6,50	Barcode ship 04 BC04 Oslo 5 timbers (Daly 2015a)
Z089m001	AD1399	AD1581	6,16	Barcode skib 5 Oslo 9 timbers (Daly 2013a)

Tabel 2. Algade 61 Tiendeladen 7, Aalborg. Resultaterne af synkroniseringsberegninger mellem middelkurve H011M001 og diverse lokal- og grundkurver. Den grå tone fremhæver de høje *t*-værdier. Kilden til kurverne er angivet.

Proveniens

I tabel 1 vises synkroniseringsberegningerne (*t*-værdi) imellem alle de undersøgte prøver fra bygningen. Årringskurverne viser generelt høj lighed, hvilket indikerer, at træerne voksede i samme region. Årringskurverne sammenregnes til en middelkurve (H011M001) på 178 år. I tabel 2 vises synkroniseringsberegningerne mellem middelkurven og et udvalg af årringsdata. De højeste korrelationer opnås med en række årringsdatasæt for Sydskandinavien. Tømmeret dateres til en periode, hvor en del tømmerhandel og transport fandt sted lige netop i denne region. Det er derfor vanskeligt at bestemme træets oprindelse meget præcist. Den højeste lighed er med et skibsvrag fra Oslo, BC14, lavet af egetræ, som sandsynligvis stammer fra Vestsverige og med et sæt årringsdata fra Gammel Strand i København, som også kan stamme fra Vestsverige.

Analysen

Datafangst og bearbejdning af materialet er foretaget med programmet ”DENDRO” (Tyers, 1997) og til beregning af *t*-værdien (synkroniseringsværdien ”*t*-test”) benyttes ”CROS” (Baillie & Pilcher, 1973). Til analysen er benyttet grund- og lokalkurver fra

Nordeuropa. Til beregning af fældningstidspunkt for det undersøgte egetræ er her benyttet en splintstatistik udarbejdet for Norge. Den viser, at egetræer har ca. 15 splintår i gennemsnit (-8 +6) (Christensen & Havemann 1998). En detaljeret beskrivelse af proveniensbestemmelsesmetoden findes bl.a. i Daly (Daly 2007).

Litteratur

Baillie, M.G.L. and Pilcher, J.R., 1973. A simple crossdating program for tree-ring research. *Tree-Ring Bulletin* 33, 7-14.

Bråthen, A. 1982. Dendrokronologisk serie från västra Sverige 813-1975. *Rapport Riksantikvarieämbetet och Statens historiska museer* 1982:1. Stockholm.

Christensen, K. & Havemann, K. 1998. Dendrochronology of oak (*Quercus sp*.) in Norway. *AmSVaria* 32, Stavanger, 59-60.

Daly, A., 1999. Dendrokronologisk undersøgelse af tømmer fra Nyborg slot, Fyns Amt. *Nationalmuseets Naturvidenskabelige Undersøgelse* rapport nr. 1999 : 25.

Daly, A., 2000a. Dendrokronologisk Undersøgelse af tømmer fra Østerå, Aalborg. *Nationalmuseets Naturvidenskabelige Undersøgelser rapport nr.* 25, 2000. København.

Daly, A., 2000b. Dendrokronologisk undersøgelse af tømmer fra B&W grunden, Skibsvrag 2 og 5. *Nationalmuseets Naturvidenskabelige Undersøgelser rapport nr.* 26, 2000, København.

Daly, A., 2001a. Dendrokronologisk undersøgelse af tømmer fra Boulevarden, Aalborg. *Nationalmuseets Naturvidenskabelige Undersøgelser* rapport nr. 2001 : 7.

Daly, A., 2001b. Dendrokronologisk undersøgelse af tømmer fra Suså, Næstved, Storstrøms amt. *Nationalmuseets Naturvidenskabelige Undersøgelser rapport nr.* 31, 2001. København.

Daly, A., 2002. Dendrokronologisk undersøgelse af tømmer fra Funder kirke, Århus amt. *Nationalmuseets Naturvidenskabelige Undersøgelser rapport nr*. 2002 : 19, København.

Daly, A., 2007. *Timber, Trade and Tree-rings. A dendrochronological analysis of structural oak timber in Northern Europe, c. AD 1000 to c. AD 1650*. Ph.D. thesis submitted February 2007, University of Southern Denmark.

Daly, A., 2009. WM2307 Gåsehage ship. *Dendro.dk rapport nr.* 2009 : 15, København.

Daly, A., 2011. Barcode vrag 5, vrag 8 og vrag 14, Oslo. *Dendro.dk rapport nr.* 2011 : 24.

Daly, A., 2013a. Barcode ship 5 BC05, Oslo. *Dendro.dk rapport* 2013 : 6.

Daly, A., 2013b. Dendrokronologiske undersøgelse af tømmer fra bolværker fra Køge Havn KNV00048. *Dendro.dk rapport nr.* 2013 : 36

Daly, A., 2015a. Dendrochronological analysis of the timbers of Barcode ship 4 (BC04), from Barcode, Oslo.Dendro.dk report 20 : 2015

Daly, A., 2015b. Dendrokronologiske undersøgelse af tømmer fra skibsvrag Klippan 2, Sverige. *Dendro.dk rapport* 2015 : 40, Copenhagen.

Daly, Aoife, 2016. Dendrokronologisk undersøgelse af tømmer fra Strandgade, Nibe NJM 6420. *dendro.dk report* 2016:10, Copenhagen.

Tyers, I.G., 1997. Dendro for Windows Program Guide, *ARCUS Report* 340, Sheffield.

Katalog

Filename	sample title and number	rings	start yr.	End yr.	pith	sapwood	bark?	Conversion	extra end	Ave. Ring width mm	interpretation / felling
samples											
H011001a	ÅHM 6465 Aalborg Algade Tiendeladen hus overgangsfodrem x75B	128	AD1429	AD1556	C	2	N	S	N	0.91	AD1564-79
H011002a	ÅHM 6465 Aalborg Algade Tiendeladen hus skråstiver x76	178	AD1386	AD1563	C	16	N	S	N	0.67	AD1563-72
H011003a	ÅHM 6465 Aalborg Algade Tiendeladen hus skråstiver x77	160	AD1404	AD1563	C	11	N	S	S4	0.64	AD1567-77
H011004a	ÅHM 6465 Aalborg Algade Tiendeladen hus tap x78	114	AD1435	AD1548	G	0	B	T	N	0.74	AD1560-75
H011005a	ÅHM 6465 Aalborg Algade Tiendeladen hus tap x79	72	AD1484	AD1555	G	5	N	T	S1	0.95	AD1560-75
Averages											
H011ST4&5	ÅHM 6465 Aalborg Algade Tiendeladen hus tap same tree x78 x79	121	AD1435	AD1555	G	5	N	T	S1	0.80	AD1560-75
H011M001	ÅHM 6465 Aalborg Algade Tiendeladen hus 4 timbers	178	AD1386	AD1563						0.73	
Conversion: R = radial split plank, T = tangential plank, W = whole timber, S = squared whole timber, H = half timber, Q = quarter timber, O = other conversion.<br>Pith: C = centre, V = less than 5 rings, F = 5 – 10 rings, G = greater than 10 rings.											
Aoife Daly, ph.d.					22 January 2016						

When quoting these results please add the following:

in publication bibliography/literature lists:	Daly, Aoife, 2016. Dendrokronologisk undersøgelse af tømmer fra Algade 61 Tiendeladen 7, Aalborg, NJM 6465. *dendro.dk report* 2016:11, Copenhagen.
In blogs and social media:	*dendro.dk report* 2016:11
