# Supplementary material for: Provenancing 16th and 17th century CE building timbers in Denmark–combining dendroprovenance and Sr isotopic analysis
Source: PLoS One. 2023 Feb 9;18(2):e0278513. doi: 10.1371/journal.pone.0278513 (PMC9910641; doi:10.1371/journal.pone.0278513)
Supplement: S2 File — Daly, A., 2020. Dendrochronological analysis of timbers from Brix Gård, Aalborg ÅHM 7235. Dendro.dk report 2020:7, Copenhagen. (PDF) [file pone.0278513.s002.pdf]

Dendrochronological analysis of timbers from Brix Gård, Aalborg ÅHM 7235

Aoife Daly
Dendro.dk report 7 : 2020
Commissioned by Christian Klinge, Nordjyllands Historiske Museum.

Two samples from one beam in a building at Brix Gård, Aalborg were submitted to dendro.dk for analysis, to determine the date and provenance of the timber. The results are described in this report.

When quoting these results please add the following:
in publication bibliography/literature lists:
Daly, A., 2020. Dendrochronological analysis of timbers from Brix Gård, Aalborg ÅHM 7235. *Dendro.dk report* 2020:7, Copenhagen.
In blogs and social media: dendro.dk report 2020:7

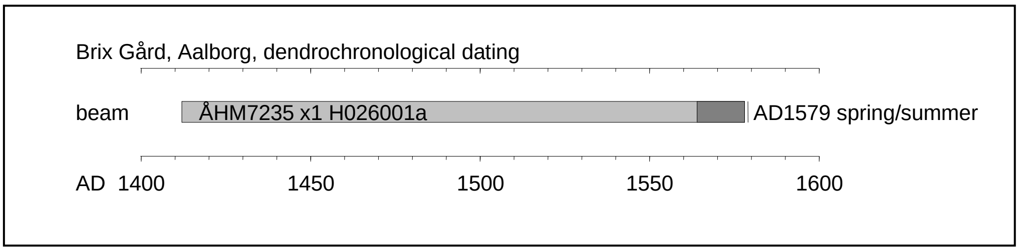

Fig. 1. Brix Gård, Aalborg. The diagram illustrates the chronological position of the dated beam.

Brix Gård, Aalborg

The beam is Quercus sp., oak and contains 167 tree-rings of which 14 are sapwood rings. The sapwood is preserved complete to bark edge and the outermost ring is not fully formed, which tells us that the tree was felled in the growing season.
The sample is dated. The tree-ring curve covers the period AD 1412 to 1578. The tree was felled in spring/summer 1579.

Provenance

In table 1 the correlation between the tree-ring curve from the beam from Brix Gård and a wide range of Northern European oak tree-ring datasets are shown. The material is dating strongly with a range of oak tree-ring datasets that represent timber from west Sweden.

Methodology

Measuring and analysis of the material is carried out using the program 'DENDRO' (Tyers, 1997) and for the calculation of the *t*-value ('*t*-test') 'CROS' (Baillie & Pilcher, 1973) is used. In the analysis master and site chronologies for Northern Europe are used.

Filenames	-	-	H026001a	
-	start	dates	AD1412	
-	dates	end	AD1578	
Master and site chronologies				
WSwedM25	AD1344	AD1560	9.10	West Sweden 49 timbers (Daly unpubl)
SScanM25	AD1343	AD1548	8.41	Southern Scandinavia 36 timbers (Daly unpubl)
8127M001	AD846	AD1771	6.57	Ålborg Østerå + Boulevarden 67 timbers (Daly 2000a 2001a)
F041M002	AD1354	AD1686	6.54	Hastrup Mølle 12 timbers (Daly 2018)
SM100001	AD1310	AD1539	6.22	Ystad area (Lund University)
4077M001	AD1310	AD1540	5.99	Nyborg slot (Daly 1999)
B037M001	AD1337	AD1550	5.99	Favrholm Mølle 20 timbers (Daly 2016e)
8111M002	AD1350	AD1480	5.90	Astrup K. 7 timbers (Daly 1998b)
6094M002	AD1450	AD1660	5.89	Funder kirke later material 4 timbers (Daly 2002)
midtjy17	AD536	AD1980	5.85	Mid Jutland (Christensen pers comm)
Chronologies from Swedish imports				
D0137M04	AD1286	AD1520	9.78	TBT Odense group4 12 timbers (Daly & Daly 2019)
H011M001	AD1386	AD1563	9.71	Aalborg Algade Tiendeladen hus 4 timbers (Daly 2016b)
B027oak B p...	AD1248	AD1532	8.83	Gammel Strand Copenhagen B purple 21 timbers (Daly 2016d)
NB700000	AD1345	AD1538	8.46	Helsingør (Nationalmuseet)
Ep3mnall	AD1361	AD1539	8.46	Stirling Castle Scotland (Crone pers comm)
q415029m04	AD1356	AD1540	8.14	Evangelistas altarpiece Seville Cathedral 29 planks (Rodríguez-Trobajo & Domínguez-Delmás 2015)
D0134M03	AD1347	AD1507	7.77	Thomas b Thriges bro 6 timbers (Daly 2017)
B027oak C o...	AD1331	AD1557	7.73	Gammel Strand Copenhagen C orange 23 timbers (Daly 2016d)
EP31539	AD1361	AD1539	7.46	Stirling Castle Scotland (Crone pers comm)
2121M002	AD1052	AD1596	7.45	Suså Næstved all posts (Daly 2001b)
H009M001	AD1410	AD1613	7.41	Strandgade Nibe bolværk A6 3 timbers (Daly 2016a)
EP41592	AD1390	AD1592	7.17	Stirling Castle Scotland episode 4 (Crone pers comm)
ZEALAND0	AD452	AD1770	7.11	Sjælland Denmark 249 timbers (Daly unpubl)
2M000006	AD1318	AD1514	7.10	Sjælland kirker (Daly e.g. 1998a)
Chronologies from shipwrecks				
Z0923M03	AD1328	AD1618	9.15	Vasa group 3 31 timbers (Daly forthcoming)
Z073m001	AD1385	AD1574	9.10	Barcode 14 ship Oslo 3 timbers (Daly 2011)
Z141M001	AD1352	AD1539	8.52	Klippan 2 shipwreck Göteborg 11 timbers (Daly 2015b)
WSshipsM01	AD1352	AD1636	7.93	West Swedish ships 29 timbers (Daly unpubl)
bispevika09&12	AD1365	AD1539	6.71	Bispevika 09 & 12 ship Oslo 2 timbers (Daly unpubl)
Z040M001	AD1386	AD1567	6.62	Gåsehage Randers 2 timbers (Daly 2009)
Z173M001	AD1354	AD1547	6.61	Bispevika 7 ship Oslo 2 timbers (Daly 2016c)
Z249M001	AD1375	AD1588	6.22	Bispevika 16 Oslo 2 timbers (Daly 2019)
00652M02	AD1405	AD1607	6.14	B&W Grund Vrag 2. 2 trees (Daly 1997; 2000b)
Z119M001	AD1317	AD1573	5.79	Barcode Oslo ship 4 BC04 5 timbers (Daly 2015a)

Table 1. Brix Gård, Aalborg. Result of the correlation between the tree-rings curve from the beam H026001a and diverse Northern European site and master chronologies. The source of the chronologies is given. The grey tone highlights the high *t*-values.

Literature

Baillie, M.G.L. and Pilcher, J.R., 1973. A simple crossdating program for tree-ring research. *Tree-Ring Bulletin* 33, 7-14.

Daly, A., 1997. Dendrokronologisk undersøgelse af tømmer fra 'B&W grunden', Strandgade 3A, Christianshavn, tidligere Grønnegaard Havn. II: Skibsvrag mm. *Naturvidenskabelige Undersøgelser rapport* 1997:5, Copenhagen.

Daly, A., 1998a. Dendrokronologisk undersøgelse af tømmer fra Ejby kirke, Roskilde Amt. *Nationalmuseets Naturvidenskabelige Undersøgelser rapport* 1998:10, Copenhagen.

Daly, A., 1998b. Dendrokronologisk undersøgelse af tømmer fra Astrup kirke, Hjørring Amt. *Nationalmuseets Naturvidenskabelige Undersøgelser rapport* 1998:30, Copenhagen.

Daly, A., 1999. Dendrokronologisk undersøgelse af tømmer fra Nyborg slot, Fyns Amt. *Nationalmuseets Naturvidenskabelige Undersøgelse rapport* 1999:25, Copenhagen.

Daly, A., 2000a. Dendrokronologisk Undersøgelse af tømmer fra Østerå, Aalborg. *Nationalmuseets Naturvidenskabelige Undersøgelser rapport* 2000:25, Copenhagen.

Daly, A., 2000b. Dendrokronologisk undersøgelse af tømmer fra B&W grunden, Skibsvrag 2 og 5. *Nationalmuseets Naturvidenskabelige Undersøgelser rapport* 2000:26, Copenhagen.
Daly, A., 2001a. Dendrokronologisk undersøgelse af tømmer fra Boulevarden, Aalborg. *Nationalmuseets Naturvidenskabelige Undersøgelser rapport* 2001:7, Copenhagen.
Daly, A., 2001b. Dendrokronologisk undersøgelse af tømmer fra Suså, Næstved, Storstrøms amt. *Nationalmuseets Naturvidenskabelige Undersøgelser rapport* 2001:31, Copenhagen.
Daly, A., 2002. Dendrokronologisk undersøgelse af tømmer fra Funder kirke, Århus amt. *Nationalmuseets Naturvidenskabelige Undersøgelser rapport* 2002:19, Copenhagen.
Daly, A., 2009. WM2307 Gåsehage ship. *Dendro.dk rapport nr.* 2009:15, Copenhagen.
Daly, A., 2011. Barcode vrag 5, vrag 8 og vrag 14, Oslo. *Dendro.dk rapport nr.* 2011:24, Copenhagen.
Daly, A., 2015a. Dendrochronological analysis of the timbers of Barcode ship 4 (BC04), from Barcode, Oslo. *Dendro.dk report* 2015:20, Copenhagen.
Daly, A., 2015b. Dendrokronologiske undersøgelse af tømmer fra skibsvrag Klippan 2, Sverige. *Dendro.dk rapport* 2015:40, Copenhagen.
Daly, A., 2016a. Dendrokronologisk undersøgelse af tømmer fra Strandgade, Nibe NJM 6420. *dendro.dk report* 2016:10, Copenhagen.
Daly, A., 2016b. Dendrokronologisk undersøgelse af tømmer fra Algade 61 Tiendeladen 7, Aalborg NJM 6465 *Dendro.dk rapport* 2016:11, Copenhagen.
Daly, A., 2016c. Dendrochronological analysis of timbers from the ship 'Bispevika 7', Oslo. *Dendro.dk report* 2016:37, Copenhagen.
Daly, A., 2016d. Dendrochronological analysis of timber from Gammel Strand, Copenhagen. *Dendro.dk report* 2016:44, Copenhagen.
Daly, A., 2016e. Dendrochronological analysis of timber from a mill at Favrholm Mølle, near Hillerød, Denmark. *Dendro.dk report* 2016:47, Copenhagen.
Daly, A., 2017. Dateringsundersøgelse af tømmer fra bro og bolværk fundet ved Thomas B. Thriges Gade, Odense. *Dendro.dk rapport* 2017:37, Copenhagen.
Daly, A., 2018. Dendrokronologisk undersøgelse af tømmer fra Hastrup Mølle VKH7870. *Dendro.dk rapport* 2018:63, Copenhagen.
Daly, A., 2019. Dendrochronological analysis of ship timbers from three wrecks Bi16, Bi17 & Bi18 at Bispevika, Oslo. *dendro.dk report* 2019:48, Copenhagen.
Daly, A. & Daly, S., 2019. Dateringsundersøgelse af tømmer fra Thomas B. Thriges Gade, Odense. *dendro.dk report* 2019:47, Copenhagen.
Rodríguez-Trobajo, E. & Domínguez-Delmás, M., 2015. Swedish oak, planks and panels: dendroarchaeological investigations on the 16th century Evangelistas altarpiece at Seville Cathedral (Spain) *Journal of Archaeological Science* 54, 148–161.
Tyers, I.G., 1997. Dendro for Windows Program Guide, *ARCUS Report* 340, Sheffield.

Catalogue

Filename	sample title and number, species	rings	start yr.	end yr.	pith	sapwood	bark?	Conversion	extra end	Ave ring width mm	Interpretation / felling
Samples											
H026001a	Brix Gård Aalborg ÅHM 7235 x1 QUSP	167	AD1412	AD1578	C	14	½s/s	S	N	1.10	AD1579 spring/summer
Conversion: R = radial split plank, T = tangential plank, W = whole timber, S = squared whole timber, H = half timber, Q = quarter timber, O = other conversion.<br>Pith: C = centre, V = less than 5 rings, F = 5 – 10 rings, G = greater than 10 rings.<br>QUSP = *Quercus sp*., oak. PISY = *Pinus sp*., pine. PCAB = *Picea sp/Larix sp*., spruce/larch. ABAL = *Abies sp*., fir.											
Aoife Daly, Ph.D.					31 March 2020						
