# Supplementary material for: Provenancing 16th and 17th century CE building timbers in Denmark–combining dendroprovenance and Sr isotopic analysis
Source: PLoS One. 2023 Feb 9;18(2):e0278513. doi: 10.1371/journal.pone.0278513 (PMC9910641; doi:10.1371/journal.pone.0278513)
Supplement: S3 File — Daly, A., 2019. Dendrokronologisk undersøgelse af tømmer fra bygning, Nørregade 12, Horsens (HOM 2393). dendro.dk report 2019:5, Copenhagen. (PDF) [file pone.0278513.s003.pdf]

Dendrokronologisk undersøgelse af tømmer fra bygning, Nørregade 12, Horsens (HOM 2393)

Aoife Daly, ph.d.
Dendro.dk rapport 5 : 2019
Indsendt af Frederik Callesen, Horsens Museum.

I denne rapport beskrives den dendrokronologiske analyse af prøver fra stolper og bjælker fra bindingsværk på Nørregade 12, Horsens.

Tretten prøver fra 11 tømmeremner fra bygningen er indsendt med henblik på dendrokronologisk dateringsanalyse.
Tre af prøverne er af *Pinus sp.*, fyr, én er af *Picea sp./Larix sp.*, gran/lærk, og de resterende er af *Quercus sp.*, eg. Én prøve, DP17 af eg, indeholder kun 41 årringe og er ikke undersøgt nærmere. Af de 12 analyserede prøver er ni dateret.

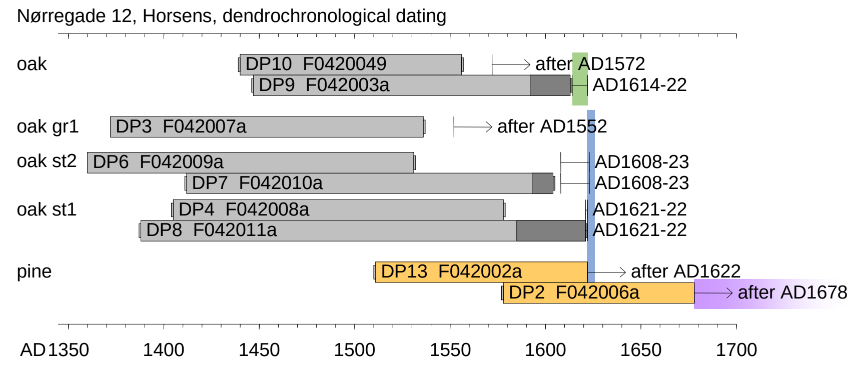

Fig. 1. Nørregade 12, Horsens. Diagrammet viser dateringen af prøverne.

Egetræsprøverne

Af de otte egetræsprøver, som er analyseret, er ni dateret. Fem af prøverne danner en gruppe, idet deres årringskurver har høj lighed. To af disse har splintved bevaret.
Prøve F042010a (DP7) som er fra samme stolpe som F042009a (DP6), har 11 splintår. Ved tillæg for manglende splintved er fældningstidspunktet for træet, som stolpen blev fremstillet af, beregnet til **1608-23 e.Kr.**
Prøve F042011a (DP8), som er fra samme stolpe som prøve F042008a (DP4), har 36 splintår. Ved tillæg for manglende splintved er fældningstidspunktet for træet, som stolpen blev fremstillet af, beregnet til **1621-22 e.Kr.**
Hvis denne gruppe repræsenterer træer, der blev fældet samtidig, kan denne fældning tidsbestemmes til **ca. 1621-22 e.Kr.** (markeret med blåt i fig. 1).

To egetræsprøver falder uden for gruppen. De krydsdaterer ikke indbyrdes, men er begge dateret særskilt. Prøve F0420049 (DP10) indeholder 117 årringe, har kun kerneved bevaret og er fra et træ fældet **efter 1572 e.Kr.** Prøve F042003a (DP9) har 167 årringe heraf 21 splintår og er fra et træ fældet **ca. 1614-22 e.Kr.** Disse to prøver kan således også høre til samme fældningsfase som de øvrige egeprøver.

En ottende egetræsprøve indeholder kun 63 årringe og er ikke dateret.

			F042002a	F042006a	F0420049	F042003a	F042007a	F042009a	F042010a	F042008a	F042011a
	Pine DP13	F042002a	*	2,4	0,43	2,15	-	-	0,04	-	-
	Pine DP2	F042006a	2,4	*	\	-	\	\	-	\	-
	Oak DP10	F0420049	0,43	\	*	0,98	0,72	0,52	0,76	0,13	0,28
	Oak DP9	F042003a	2,15	-	0,98	*	0,64	1,09	1,41	0,39	0,41
Average F042M001	Oak DP3	F042007a	-	\	0,72	0,64	*	6,26	4,53	5,4	7,65
	Oak DP6 Oak DP7 same tree	F042009a	-	\	0,52	1,09	6,26	*	10,33	7,3	9,48
		F042010a	0,04	-	0,76	1,41	4,53	10,33	*	7,5	8,18
	Oak DP4 oak DP8 same tree	F042008a	-	\	0,13	0,39	5,4	7,3	7,5	*	16,05
		F042011a	-	-	0,28	0,41	7,65	9,48	8,18	16,05	*

Tabel 1. Nørregade 12, Horsens. Resultaterne af synkronisering, internt. Den grå tone fremhæver de høje t-værdier.

Filenames	-	-	F042M001	
-	start	dates	AD1360	
-	dates	end	AD1621	
9M456781	109BC	AD1986	7.52	Jylland/Fyn (Nationalmuseet)
CD60NZ01	AD1377	AD1576	6.37	Skafågård 12 timbers (Nationalmuseet revised Daly 2007)
CD51JZ03	AD1346	AD1497	5.95	Møllestrømmen 3 timbers (Nationalmuseet revised Daly 2007)
G008M001	AD1344	AD1493	5.94	Skodborghus Møllebakken 11 timbers (Christensen pers comm)
H137PM01	AD1408	AD1555	5.89	Seeth Haus 3 timbers (Hamburg Uni revised Daly 2007)
CD51MZ01	AD1364	AD1585	5.78	Gram Bro 22 timbers (Nationalmuseet revised Daly 2007)
H131YM01	AD1409	AD1575	5.62	Herrenhaus Ostaerrad 11 timbers (Hamburg Uni revised Daly 2007)
H115CM01	AD1452	AD1674	5.50	Preetz Markt 24 9 timbers (Hamburg Uni revised Daly 2007)
CD50PZ01	AD1285	AD1482	5.45	Varns Klokkeh 3 timbers (Nationalmuseet revised Daly 2007)
Z0921M03	AD1341	AD1625	5.39	Vasa mostly frames 9 timbers (Daly forthcoming)
H11ECM01	AD1368	AD1502	5.33	St.Johannis Klost 5 timbers (Hamburg Uni revised Daly 2007)
4077M002	AD1396	AD1542	5.31	Nyborg slot 3 trees (Daly 1999)
H12A1M01	AD1396	AD1541	5.28	Lunden. Hof Eiberg 5 timbers (Hamburg Uni revised Daly 2007)
CD60JZ01	AD1385	AD1652	5.22	Ulstrup 3 timbers (Nationalmuseet revised Daly 2007)
G312NZ01	AD1413	AD1576	5.19	Bevern 3 timbers (Göttingen Uni revised Daly 2007)
H11JXM01	AD1385	AD1451	4.94	HL-Koberg 2 6 timbers (Hamburg Uni revised Daly 2007)
4077M003	AD1418	AD1546	4.90	Nyborg slot 2 trees (Daly 1999)
H11HHM01	AD1379	AD1531	4.87	HL Langer Lohberg 47 14 timbers (Hamburg Uni revised Daly 2007)
CD51JZ02	AD1401	AD1502	4.86	Møllestrømmen 4 timbers (Nationalmuseet revised Daly 2007)
G330OZ01	AD1391	AD1482	4.86	Hildesheim 14 timbers (Göttingen Uni revised Daly 2007)
CD60OZ01	AD1370	AD1588	4.85	Bidstrup 5 timbers (Nationalmuseet revised Daly 2007)
H129JM01	AD1449	AD1616	4.84	Jersbek 9 timbers (Hamburg Uni revised Daly 2007)
H11KLM01	AD1385	AD1589	4.48	HL Mengstr. 44 18 timbers (Hamburg Uni revised Daly 2007)
DM100008	AD457	AD1723	4.18	Lübeck (Hamburg Uni)

Tabel 2. Nørregade 12, Horsens. Resultaterne af synkroniseringsberegninger mellem middelkurven F042M001 og diverse lokal- og grundkurver. Den grå tone fremhæver de høje t-værdier. Kilden til kurverne er angivet.

Filenames	-	-	DP9 F042003a	DP10 F0420049	
-	start	dates	AD1447	AD1440	
-	dates	end	AD1613	AD1556	
F012M001	AD1456	AD1574	6.25	-	Gl. Estrup voldgrav 2 timbers (Daly unpubl)
midtjy17	AD536	AD1980	5.73	4.95	Mid Jutland (Christensen pers comm)
B012M001	AD1347	AD1484	4.92	-	Copenhagen Admiralgade 3 timbers (Daly 2005)
CD60NZ01	AD1377	AD1576	4.85	-	Skafågård 12 timbers (Nationalmuseet revised Daly 2007)
9M456781	109BC	AD1986	4.79	5.20	Jylland/Fyn (Nationalmuseet)
Z1080M02 ship2	AD1358	AD1618	4.54	-	Cuxhaven Ship BSH 9834 ship group2 9 timbers (Daly 2014)
CD60JZ01	AD1385	AD1652	4.23	5.70	Ulstrup 3 timbers (Nationalmuseet revised Daly 2007)
CD60HZ01	AD1341	AD1551	-	6.00	Sostrup 6 timbers (Nationalmuseet revised Daly 2007)
PP11203A	AD1447	AD1570	-	5.03	PL Gdansk-Lipce (Wazny pers comm)
CD60OZ01	AD1370	AD1588	-	4.88	Bidstrup 5 timbers (Nationalmuseet revised Daly 2007)

Tabel 3. Nørregade 12, Horsens. Resultaterne af synkroniseringsberegninger mellem årringskurverne fra egetræsprøverne F042003a (DP9) og F0420049 (DP10) og diverse lokal- og grundkurver. Den grå tone fremhæver de høje t-værdier. Kilden til kurverne er angivet.

Fyrretræsprøverne
To af de tre fyrretræsprøver kunne dateres. Prøve DP15 (F042001a) indeholder kun 60 årringe og er ikke dateret. Prøve DP13 (F042002a) har 112 årringe og er dateret. Træet, som prøven kommer fra, er fældet **efter 1622 e.Kr.** DP13 kan således være samhørende med egetræsfasen som er fra ca. 1621-22 e.Kr. (markeret med blåt på fig. 1).
Prøve F042006a (DP2) har 101 årringe og er dateret. Træet, som prøven kommer fra, er fældet **efter 1678 e.Kr.**

Gran-/lærketræet
En enkelte prøve er af gran-/lærk og indeholder 71 årringe. Prøven kunne ikke dateres.

Filenames	-	-	DP13 F042002a	DP2 F042006a	
-	start	dates	AD1511	AD1578	
-	dates	end	AD1622	AD1678	
Scandinavian pine chronologies					
N035m6_k8	AD1455	AD1595	6.23	-	Oslo Bjørvika B2 k8 5 timbers (Daly 2017b)
NOMK0803	AD1345	AD1780	5.94	4.35	Norway Aust Agder (Bartholin pers comm)
N0254M01	AD1421	AD1570	5.33	\	Oslo B3B7 10 timbers (Daly 2017a)
20000059	AD1488	AD1647	5.29	6.08	Oslo Revierstr (Bartholin pers comm)
N0253M01	AD1351	AD1621	4.54	7.67	Oslo Bispevika B3B7 61 timbers (Daly 2016b)
Chronologies of pine exported to elsewhere in Europe					
BHIMNx5	AD1401	AD1583	7.12	\	Scotland pine BHMNx5 (Crone pers comm)
IMPORTx8	AD1329	AD1671	6.26	4.62	Scotland pine imports 59 timbers (Crone pers comm)
gbl01	AD1504	AD1719	6.17	-	London Camden St Georges Church Bloomsbury (Bridge pers comm)
k010301s	AD1395	AD1706	5.92	5.83	Gulfhauser farm Lower Saxony Swedish timber (Crone pers comm)
N007m005	AD1471	AD1622	5.84	5.71	Barcode 11-13 Oslo Bolværk 22 timbers (Daly 2008)
ToLQHS2	AD1497	AD1677	5.80	5.39	Queens House Tower of London pine 3 timbers (Bridge pers comm)
FORTHx6	AD1329	AD1619	5.72	-	Scotland pine FORTHx6 38 timbers (Crone pers comm)
SPPINEx15	AD1476	AD1671	5.11	4.75	Scotland pine Stirling Palace beams (Crone pers comm)
FYRSVEN3	AD1353	AD1636	5.00	6.79	Svendborg pine (Bartholin pers comm)
B027pine C .	AD1484	AD1642	4.88	5.62	Gammel Strand Copenhagen C orange 5 timbers (Daly 2016a)
73KG-B	AD1551	AD1699	3.81	5.14	Kew Green Richmond Surrey (C Tyers pers comm)
X003M001	AD1299	AD1687	3.35	4.97	Jarssum Emden East Frisia dyke all 5 timbers (Daly 2013)
21015M02	AD1305	AD1743	-	5.24	B&W grund 24 trees (Daly 1997a & b)

Tabel 4. Nørregade 12, Horsens. Resultaterne af synkroniseringsberegninger mellem årringskurverne fra fyrretræsprøverne F042002a (DP13) og F042006a (DP2) og diverse lokal- og grundkurver. Den grå tone fremhæver de høje t-værdier. Kilden til kurverne er angivet.

Proveniens

I tabel 1 vises synkroniseringsberegningerne årringskurverne imellem. Prøverne DP6 & DP7 er fra samme stolpe og er sammenregnet til én årringskurve (F042009&10 st). Prøverne DP4 & DP8 er også fra samme stolpe, og de er også sammenregnet til én årringskurve (F042008&11 st). En middelkurve (F042M001) på 262 år er beregnet ud fra de tre træer i gruppen (fremhævet med blåt i tabel 1).
Synkroniseringen mellem middelkurven og et udvalg af årringsdata vises i tabel 2. Den opnår højeste korrelation (t-værdi) med grund- og lokalkurver for Sønderjylland.

Synkronisering mellem prøverne DP9 (F042003a) og DP10 (F0420049) og diverse grund- og lokalkurver vises i tabel 3. Begge viser også bedste lighed med jyske lokalkurver.

Synkronisering mellem de to daterede fyrretræsprøver og diverse grund- og lokalkurver vises i tabel 4. Begge viser højeste lighed med norske grundkurver, samt med en række lokalkurver bygget af skandinavisk eksporttræ.

Analysen

Datafangst og bearbejdning af materialet er foretaget med programmet ”DENDRO” (Tyers, 1997) og til beregning af *t*-værdien (synkroniseringsværdien ”t-test”) benyttes ”CROS” (Baillie & Pilcher, 1973). Til beregning af fældningstidspunktet for egetræet, er splintstatistik for Nordtyskland benyttet: ca. 20 (-5 +10) (Hollstein 1980). Splintved på fyrretræ varierer en del, og er også vanskeligt at bestemme med sikkerhed, så hvis ikke barkkant er bevaret på en fyrre- eller granprøve så er fældningstidspunkt sat til efter den yngste bevarede årring.

Litteratur

Baillie, M.G.L. and Pilcher, J.R., 1973. A simple crossdating program for tree-ring research. *Tree-Ring Bulletin* 33, 7-14.

Daly, A., 1997a. Dendrokronologisk undersøgelse af tømmer fra ‘B&W grunden’, Strandgade 3A, Christianshavn, tidligere Grønnegaard Havn. I: Bolværk, bedding mm. *Naturvidenskabelige Undersøgelser rapport* 1997:1, Copenhagen.

Daly, A., 1997b. Dendrokronologisk undersøgelse af tømmer fra ‘B&W grunden’, Strandgade 3A, Christianshavn, tidligere Grønnegaard Havn. III: Bolværk. *Naturvidenskabelige Undersøgelser rapport* 1997:18, Copenhagen.

Daly, A., 1999. Dendrokronologisk undersøgelse af tømmer fra Nyborg slot, Fyns Amt. *Nationalmuseets Naturvidenskabelige Undersøgelse rapport* 1999:25, Copenhagen.

Daly, A., 2005. Dendrokronologisk undersøgelse af træ fra Admiralgade, Copenhagen. *dendro.dk rapport* 2005:1, Copenhagen.

Daly, A., 2007. *Timber, Trade and Tree-rings. A dendrochronological analysis of structural oak timber in Northern Europe, c. AD 1000 to c. AD 1650*. Ph.D. thesis submitted February 2007, University of Southern Denmark.

Daly, A., 2008. Barcode 11-13, Oslo, Norge. *Dendro.dk report* 2008:31, Copenhagen.

Daly, A., 2013. Dendrochronological analysis of a coastal dike at Jarßum, near Emden, East Frisia, Lower Saxony, Germany. *Dendro.dk report* 2013:27, Copenhagen.

Daly, A., 2014. Dendrochronological analysis of ship timbers and timber cargo of the Elbe Wreck, Cuxhaven, Germany. *Dendro.dk report* 2014:6, Copenhagen.

Daly, A., 2016a. Dendrochronological analysis of timber from Gammel Strand, Copenhagen. *Dendro.dk report* 2016:44, Copenhagen.

Daly, A., 2016b. Dendrokronologisk undersøgelse (fase 2) af tømmer fundet ved Bispevika (B3/B7) Oslo, Norge. *Dendro.dk rapport* 2016:64, Copenhagen.

Daly, A., 2017a. Dendrokronologisk undersøgelse af yderligere tømmer (fase 3) fundet ved Bispevika (B3/B7) Oslo, Norge. *Dendro.dk rapport* 2017:28, Copenhagen.

Daly, A., 2017b. Dendrochronological analysis of timber found at Bjørvika B2 Oslo, Norway. *Dendro.dk report* 2017:48, Copenhagen.
Hollstein, E., 1980. *Mitteleuropäische Eichenchronologie.* Trierer Grabungen und Forschungen 11, Mainz am Rhein.
Tyers, I.G., 1997. Dendro for Windows Program Guide, *ARCUS Report* 340, Sheffield.

Catalogue

Filename	sample title and number, species	rings	start yr.	end yr.	pith	sapwood	bark?	Conversion	extra end	Ave ring width mm	Interpretation / felling
Samples											
F042001a	HOM 2393 Nørregade 12 3 DP15 REM PISY	60			C	0	N	O	H1	1,71	undated
F042002a	HOM 2393 Nørregade 12 4 DP13 Stolpe PISY	112	AD1511	AD1622	C	0	N	O	N	1,16	after AD1622
F042003a	HOM 2393 Nørregade 12 9 DP9 Rem QUSP	167	AD1447	AD1613	F	21	N	O	S1	0,83	AD1614-22
F0420049	HOM 2393 Nørregade 12 DP10 QUSP	117	AD1440	AD1556	F	0	N	O	H1	1,40	after AD1572
F042005a	HOM 2393 Nørregade 12 DP1 PCAB	71			C	0	N	S	H1	1,72	undated
F042006a	HOM 2393 Nørregade 12 DP2 PISY	101	AD1578	AD1678	C	0	N	S	N	1,44	after AD1678
F042007a	HOM 2393 Nørregade 12 DP3 QUSP	165	AD1372	AD1536	V	0	N	O	H1	1,00	after AD1552
F042008a	HOM 2393 Nørregade 12 DP4 QUSP	174	AD1405	AD1578	F	0	N	O	H1	0,99	AD1621-22
F042009a	HOM 2393 Nørregade 12 DP6 QUSP	172	AD1360	AD1531	F	0	N	O	H1	1,45	AD1608-23
F042010a	HOM 2393 Nørregade 12 DP7 QUSP	193	AD1412	AD1604	F	11	N	O	S1	1,20	AD1608-23
F042011a	HOM 2393 Nørregade 12 DP8 QUSP	234	AD1388	AD1621	F	36	N	O	S1	1,03	AD1621-22
F042012a	HOM 2393 Nørregade 12 DP14 QUSP	63			C	12	N	O	S1	2,40	Undated
Same tree											
F042008&11 st	HOM 2393 Nørregade 12 DP4&DP8 QUSP	234	AD1388	AD1621	F	36	N	O	S1	1,00	AD1621-22
F042009&10 st	HOM 2393 Nørregade 12 DP6&DP7 QUSP	245	AD1360	AD1604	F	11	N	O	S1	1,32	AD1608-23
Averages											
F042M001	HOM 2393 Nørregade 12 3 timbers QUSP	262	AD1360	AD1621						1,18	
Conversion: R = radial split plank, T = tangential plank, W = whole timber, S = squared whole timber, H = half timber, Q = quarter timber, O = other conversion.<br>Pith: C = centre, V = less than 5 rings, F = 5 – 10 rings, G = greater than 10 rings.<br>QUSP = *Quercus sp*., oak. PISY = *Pinus sp*., pine. PCAB = *Picea sp/Larix sp*., spruce/larch. ABAL = *Abies sp*., fir.											
Aoife Daly, Ph.D.				9th January 2019							

When quoting these results please add the following:
in publication bibliography/literature lists:
Daly, Aoife, 2019. Dendrokronologisk undersøgelse af tømmer fra bygning, Nørregade 12, Horsens (HOM 2393). *dendro.dk report* 2019:5, Copenhagen.
In blogs and social media: *dendro.dk report* 2019:5
