# Supplementary material for: Provenancing 16th and 17th century CE building timbers in Denmark–combining dendroprovenance and Sr isotopic analysis
Source: PLoS One. 2023 Feb 9;18(2):e0278513. doi: 10.1371/journal.pone.0278513 (PMC9910641; doi:10.1371/journal.pone.0278513)
Supplement: Attachment — Submitted filename: Review_Van Ham-Meert_&_Daly_2022.pdf [file pone.0278513.s005.pdf]

Review (anonymous)

Provenancing 16th and 17th century building timbers in Denmark – combining dendroprovenance and Sr isotopic analysis

Alicia Van Ham-Meert, Aoife Daly

Preliminary remark

Underlining and bold text passages in quotations are from the reviewer.

General statements about the text

The authors focus their research to an important topic in the historical sciences, the determination of the origin of historical timber (dendroprovenancing). They enter new scientific territory by extending the conventional procedure in dendrochronology of comparing tree-ring width curves of the timbers to be determined in their origin with contemporaneous regional tree-ring calendars by comparing the strontium isotope signatures of the timbers with the Sr signatures of potential growth areas. This is an innovative approach suitable to test results of conventional dendroprovenancing with a different methodological approach.

Such multi-proxy analyses have long been used in the natural sciences to explore a scientific question from different methodological perspectives. A strontium isotope analysis (SIA) in the context of dendroprovenancing has come to the attention of the reviewer for the first time with this paper.

In their paper, the authors explain the chemical-technical basics, procedures and limitations (unusability of waterlogged timbers) of SIA in great detail. This is to be highly commended because it also introduces readers with less scientific education, for example from the humanities, to the subject matter, possibilities and limitations of the process. Likewise, the source-critical comments on the origin of the samples, both of the timber to be examined for its origin and of the samples from the potential localities of origin (soil, water, wood and leaf samples), which are repeatedly interspersed in the text, are a benefit for the critical reader. With regard to the strontium isotope ratios of the soil samples, changes over time, e.g. due to anthropogenic influences (agriculture), are also addressed. The description of the political conditions and borders in southern Scandinavia during the period under investigation in the 16th and 17th centuries CE is also a good contribution to the source-critical historical view of the investigations presented here. Finally, the discussion of the data in the section "Results - Baseline data" is exemplary.

Formal suggestions

Because a complementary method to dendroprovenancing is being tested here, this term should also appear in the keywords.

The years should be supplemented in the title ("*Provenancing 16th and 17th century CE ...*") and in the continuous text by the information "CE" (formerly "AD"), so that the chronological classification is unquestionable for readers of all disciplines. The religion-related "AD" (*anno domini*) should also be replaced in Figure 1 by "CE", which is now common in the natural sciences.

Chemical formulae should be resolved in subsequent brackets when first mentioned in the running text, e.g. "NH_3NO_3 *(ammonium nitrate)*", or listed as a separate list/table at the end of the article.

It is not clear whether the tree ring width curves of the wood samples are single measurements of one radius or mean curves from several radii. I think it is methodologically important to specify this. If one follows the information in the S4-file (S4 file Van Ham-Meert & Daly 2022.fh), which contains the data of the tree ring widths in the so-called Heidelberg format, it seems to be exclusively a matter of single radii. If this is the case, I consider this methodologically questionable as far as the

dendrochronological dating of the timbers is concerned. It would also be useful if these tree-ring width data in the *.fh file contained information about the "location". Currently, besides the tree ring widths, they only contain information on dating, number of tree rings, species, pith, sapwood rings and keycode (see following example).

```
HEADER:
DateEnd=1556
Length=128
Species=QUSP
Pith=1
SapWoodRings=2
KeyCode=H011001A
DATA:Tree
 149  113  113   99   81   77   67   77   79   53
  76   59   69   69   82   49   63   44   70   80
  60   73   70   54  103   86   94   75   73   68
  83   67   61   56   59   52   53   46   66   72
  58   43   45   45   44   63   71   72   56   56
  38   57   33   53   67   49   43   49   68   65
  49   45   32   29   47   55   52   65   60   54
  57   57   78   63   84   92  159  117  146  144
 139  143  130  155  168  135  175  151  144  131
  89   63   60  120   82   76  133  150  130  113
  95  133  112  138  136   88  113  126  115   98
 106  115  112  121  114  105  133  123  133  155
 104   96  112   80  188  234  262  217    0    0
```

In the text and in the illustrations, t-values are repeatedly mentioned to express the level of the dendrochronological correlation of the timbers to each other. Unfortunately, nowhere is it mentioned whether these values were calculated according to Student (1908), Baillie/Pilcher (1973) or Hollstein (1980). Also, values are described as significant or non-significant without explaining which thresholds the authors use for this.

Sometimes the information on the unit of measurement is added to the values without a space ("*... 500mg ...*"), sometimes there is a space before it ("*... 200 µL ...*"). The spelling should be consistent according to the guidelines of PLOS ONE.

Under "***Materials and Methods - Sites***" it should be mentioned that the sapwood statistics are those for oaks, and that the Brix Gård site is not mentioned there because it is bark edge wood.

For the Tiendeladen site it should be mentioned that the best correlating chronology for groups 1 and 2 comes from Gammel Strand **C**, because there are three chronologies from Gammel Strand (B, C and E) in Table 1. Here, too, significance is mentioned at the end of the section without discussing at which t-value the authors apply significance ("*However, for both these groups the correlation with the few Western Swedish chronologies (i.e. with samples found inside Sweden) is less significant.*").

For non-dendrochronologists, it can also be confusing when, on the one hand, t-values above 10 are considered best dates in the text and, on the other hand, samples H4 and H5 are considered to be from the same stem (stem-matched) because the t-value is above 10.

The heading of **Table 1** is "*Correlation between the three tree-ring groups in this study that dendrochronologically indicate a western Swedish provenance.*" But it is probably not the correlations between the three tree-ring groups, but the correlations of the individual groups to the "*Master and site chronologies*" and the "*Chronologies from ships*". This should be reworded. It should also be mentioned <u>in all tables</u> that the values given are t-values according to either Student (1908) or Baillie/Pilcher (1973) or Hollstein (1980).

In the continuous text, one should consistently repeat the t-values from the tables in brackets and name the chronologies completely. This makes it easier to understand the argumentation, **e.g. at the Brix Gård site**, where it says „*The dendrochronological correlations show highest agreement with material from Odense, Copenhagen [41] and Helsingør (table 1) but also with the group 3 timber from*

the Vasa ship [8] and a ship from Oslo (Barcode 14 [58]) both of which are probably from western Sweden."

My suggestion would be: *The dendrochronological correlations show highest agreement with material from Odense (TBT group 4, 9.78), Copenhagen (Gammel Strand B [41], 8.83) and Helsingør (8.46) (table 1) but also with the group 3 timber from the Vasa ship [8] (9.15) and a ship from Oslo (Barcode 14 [58], 9.10) both of which are probably from western Sweden.*

In Table 3 for the site Nørregade 12, Horsens, a chronology "*Jutland/Funen*" is mentioned, which were named "*Jylland or Fyn*" in the continuous text above ("*These five samples best match a chronology for Jylland or Fyn (table 3).*"). For a reader unfamiliar with the geographical designations of Denmark, such inconsistencies can be irritating. Thus, all the designations in the Supporting Information should be compared with those in the text.

The heading of Table 4 is "*Sample locations and description in Sweden.*". Here I would change the heading of the fourth column "*Sr*" to "*Sr-sample sources*". In the accompanying text, it should be explained why the sampling of the four individual locations is inconsistent in type and extent (there are surely good reasons for that).

The chemical formulae used **in the section "Sr isotopic analysis - Sr leaching and sample digestions**" I would, as already said in the introduction, either immediately dissolve in brackets, e.g. "*HNO_3 (nitric acid)*", or dissolve all the formulae used in a table. In this section it also says *"... these steps sometimes need to be repeated a number of times.*" Why is that?

In the section "Results - "Baseline data", the first paragraph reads "... (*17**x** times more in the case of Torskog 2 and 4 times more in the case of Lödöse).*" The "**x**" as a multiplication sign is probably a remnant of an earlier version of the text.

It is a little irritating that the values of the isotope ratios in Table 5 are given with 6 decimal places, but in the running text only 4 or 3 decimal places are mentioned. *„The two samples from the Gota river (LEH201 and LEH2O2) have the highest Sr isotopic signature (a range of 0.7242-0.7246 is obtained when the measurement error is taken into account)."* If we use Table 5 to calculate the measurement error (twofold standard deviation), we obtain more precise values (0.724187-0.724625) and recognise that the authors have rounded up or down. This is probably due to better clarity.

In **Tables 5 and 6**, the heading of column 4 should be named "*± 2sd*" instead of "*2sd*". This would make it clearer that a twofold standard deviation is meant here. In Table 5, the country name "*Sweden*" should be added to the heading, and in Table 6, "*Denmark*" should be added: *„Table 5: Results from Sr isotopic determination of samples along the Gota river, Sweden.", "Table 6: Sr isotopic composition of timbers from 3 buildings in Denmark."*

In the section "**Buildings**", a number error has crept into the first line: *„Four of the 5 samples from Tiendeladen cluster together $^{87}Sr/^{86}Sr$ = 0.175896-0.716879, only H4 has a slightly higher signature 0.722123 ± 0.000039 (table 6)."* It must read *"... $^{87}Sr/^{86}Sr$= 0.715895-0.716879 ..."*.

A small mistake has been made in the caption to **Figure 2**: *„Figure 2. Summary of the results of the strontium isotope analysis for the timber from the three buildings in this study, placed together with the map reproduced from Hoogewerff **et.**al. [71], fig 6. (2 column figure)*". Should read "*... from Hoogewerff **et** al. [71], ...)*

Figure subheadings or headings (figures, tables) should end consistently either with or without a full stop.

Proposals regarding content

The claim made in the **abstract** "*By adding the Sr isotopic analysis, a far more detailed interpretation of the origin of these timbers can be presented*" cannot be confirmed in the paper. The authors

themselves contradict this claim in their **conclusion** when they write, for example: "*Dendrochronology provided more precise provenance for samples F7-F11 than Sr isotopes could.*"

The problem that reference curves/master chronologies were often constructed from timbers from different regions, and that for some historical reference curves it is not possible to know exactly from which regions the timbers originate, should be made clearer with 2-3 sentences (e.g. Ernst Hollstein and Bernd Becker have integrated sample material from each other's working area for certain weakly replicated periods).

The phenomenon of timber transport/trading has not only been a problem of dendrochronology and dendroprovenancing since the mid-14th century CE. In the chapter "***Timber trade in Southern Scandinavia***" it is correctly stated that "*Gradually, from around the **mid-14th century** onwards, wood and timber in Northern Europe was traded from regions with more abundant forests to regions where these materials were in high demand and no longer locally available.".* Elsewhere ("***Danish buildings with varied timber sources***") it then irritatingly states "*From the **mid-13th century** onwards, oak was shipped from the regions south and east of the Baltic Sea, in the form of boards of varying sizes.*". Apart from these incongruent chronological approaches (mid-13th vs. mid-14th century CE), it would also be desirable for less historically educated readers to point out that extensive timber transport already took place in the Roman Empire (here Roman Imperial Period to Late Antiquity, c. 27 BCE to mid-6th century CE). There is an extensive literature on this, most recently for example

> Bernabei, M., Bontadi, J., Rea, R., Büntgen, U., Tegel, W. (2019): Dendrochronological evidence for long-distance timber trading in the Roman Empire. PLoS ONE 14(12): e0224077. https://journals.plos.org/plosone/article?id=10.1371/journal.pone.0224077

One could also briefly mention that supra-regional timber transport in prehistoric times in Europe up to the Roman period is not a problem for provenance research on timbers: the place where the timbers processed by humans were found largely corresponds to the place where the trees grew. This changes temporarily in Roman times, but to my knowledge has not yet been documented for the subsequent early and high Middle Ages (mid-6th century to mid-13th century CE). From the Late Middle Ages onwards (mid-13th century CE), the transport of timber in Europe resumed.

The sentence following Table 3, "*Using extensive tree-ring datasets across the region, and examining the highest correlations geographically, <u>the region where the timber achieves highest correlation can be pinpointed as the region where the tree grew.</u>*", I would add to the content <u>that ecologically similar growth conditions are possible in different regions</u> and can lead to similar growth curves (tree-ring width data). This is a critical point in dendrochronological provenance determination (dendroprovenancing), and it should at least be mentioned, because it cannot be ruled out that comparative data are not available from all regions and that one therefore only recognises similarities with the areas already researched, which do not necessarily have to be the region of origin.

The sentence „*Hence, we want to use strontium isotopic analysis to be able to pinpoint the source of the timber, <u>not just where it was used, but where the trees grew</u>.*" does not seem logical to me because the SIA is not supposed to clarify the place of use.

In the section "*Sr isotopes along the Gota-river*" the colleagues mentioned should also be named: "*This data completes earlier datasets collected by colleagues*". Which colleagues are these?

The problem of (undesirable) contamination of oak leaves by rainwater leaching should already be addressed in the following sentence and not further down in the text: „*Oak tree leaves were brown and collected at the foot of oak trees in forested areas.*" **In the section "Results - Baseline data"** that follows below, it then states quite correctly "*The dead leaves probably had leached some of their Sr and some Sr was replaced by less radiogenic Sr contained in rainwater.*"

In the section “**Combining the analysis techniques - …**” it says “*One of the potential biases in the method is the use of a dataset that in itself has a history of transport and re-use. This necessitates careful interpretation of dendrochronological provenance results in order to avoid circular arguments.*” Here it would be appropriate to also refer in the text to the need for the construction of "clean" regional and also supra-regional chronologies in which the origin of all wood samples is known. This is, after all, precisely the problem discussed in the essay, which the SIA is supposed to help with.

In the following sentence „*Using strontium isotopic analysis to interrogate key questions of the dendrochronological dataset an extra level of* <u>*accuracy*</u> *is achieved*”, I would phrase it more neutrally like this: “Using strontium isotopic analysis to interrogate key questions of the dendrochronological dataset an extra level of <u>information</u> is achieved”. The accuracy is not really that much better, as the authors themselves write in the following "**Conclusion**". Rather, added value is achieved through the additional safeguarding with an independent proxy, which, however, is only possible for dry woods. (see passages underlined below): „*It showed* <u>*the added value of Sr isotopic analysis on dry building timbers*</u> *as illustrated in the case study of Brix Gård.* <u>*Dendrochronology provided more precise provenance for samples F7-F11 than Sr isotopes could.*</u> *Whereas the analysis of sample F3 prompted a re-examination of the dendrochronological data to a Southern Swedish rather than Danish provenance. For the pine samples the dendrochronology identified them as originating from two locations in Norway. Sr isotopic analysis* **confirmed t*hat the two pines had different provenances***”. I would make the last part of the sentence, in bold, more neutral: „*Sr isotopic analysis* ***revealed that the two pines had different*** <u>***Sr-signatures supporting the dendrochronological results of different provenances***</u>.”
