# Supplementary material for: Provenancing 16th and 17th century CE building timbers in Denmark–combining dendroprovenance and Sr isotopic analysis
Source: PLoS One. 2023 Feb 9;18(2):e0278513. doi: 10.1371/journal.pone.0278513 (PMC9910641; doi:10.1371/journal.pone.0278513)
Supplement: Attachment — Submitted filename: 2022-06 Reply to reviewers.docx [file pone.0278513.s006.docx]

**Reply to reviewers**

**Reviewer 1**

**Abstract**
 What do you mean with “a number of case studies”. Do you mean the three buildings?

Yes, this was adapted.

Why are the last two sentences separated from the other text? Additionally, these two sentences are about what you performed. I would expect that the last sentence should be an outlook or recommendation.

They are separated, because though they helped in interpreting the Sr isotopic data from the buildings, they represent a different part of the research and trying to add that to the sentences above made the whole abstract more difficult to read.

Keywords should be better selected. I think that “16th and 17th centuries” are not the best keywords; moreover, you have it in both title and abstract. I would suggest substituting it by “historical construction”, “timber transport” or “cultural heritage”.

We thank the reviewer for their comment, however for historians such key words are useful, whereas cultural heritage is extremely broad, we’ve added construction timber as a key word.

The Introduction is too long (almost 8 pages) compared to results (~2 pages) and discussion (~2 pages), includes a lot of redundant information and lacks references. This part should be significantly shortened and thoroughly described. Redundant information should be removed and only relevant text should be preserved so that it is straightforward and follows a line of the story. I would suggest making an outline (what you want to say) and following it. There are a lot of different examples which are described in too a detail (e.g. why is it important for this study that the excavations at Gammel Strand where carried out by the Museum of Copenhagen and led by Stuart Whatley… both certainly deserve recognition but this paper is not about their activities…- this redundant information should not be in this paper). I think that only a few examples which are the most relevant for this study should be briefly mentioned with relevant references. If readers are interested, they will find more details in the literature.

We summarized the examples of Sr isotopic analysis in a more concise way. We kept them all, because this is actually an exhaustive list and shows how little this method has been applied to wood so far, whereas it is ubiquitous in the study of other archaeological remains.

We understand the reviewer’s concern on detailing everything on sites, but this is actually the only way for readers to know which samples we are talking about. Sites are often excavated by various teams over long periods of time, it is hence customary to be as precise as possible about the campaign discussed.

Why do you describe Strontium in detail? I would expect such a detailed description in a paper on biogeochemistry or maybe ecophysiology. Moreover, is it really important to know that it is “just below calcium in the periodic table”? Maybe for chemists or students but definitely not here.

The sentence was removed. However, since this is a very broad journal with a non-specialist audience it is important to give the basic information needed to understand the principles behind using Sr isotopic analysis for provenancing of timbers. We tried to shorten some of the explanation as well.

After the 3rd sentence, you refer to 8 papers but then in the following 2.5 paragraphs you only have two references. However, the references are needed there, for example - there must be various dendro studies which led you to this statement: Through dendrochronological analysis of the remains of timber structures, objects that survive in historic buildings and collections, and that are found through archaeological excavation, a detailed mapping of the sources and destinations for timber has been possible. Another example, I think that you should cite several papers here from various European regions: Master and site chronologies for many regions, from Ireland to Estonia, from Norway to Spain, that have been built from thousands of tree-ring analyses over many decades of dendrochronology in Europe.

A couple of references have been added

Extensive tests for Sr isotopic analysis of timbers from shipwrecks and other waterlogged wood were carried out in this project, and by others. Who are the “others”? Again, reference needed. I can find many examples in the Introduction. You should carefully go through it and cite relevant papers or books which you were inspired by.

The references for those “others” were at the end of the next sentence, we’ve added them at the end of this sentence as well.

“These had never been submerged, hence any Sr in the wood should reflect the geology of the place where these trees originally grew.” How do you know this? You concluded that some timbers were transported. How? In the past, timber was frequently transported on water…

Timber was floated on water, and transported by boat, but it was not submerged like for the shipwrecks or tests performed in the lab. The problem when the wood spends long a couple of weeks in water is that the indigeneous Sr is completely replaced by exogeneous Sr from the waterlogging environment: the sea, the wet ground, … In buildings, even if the original timbers were floated this is not the case.

You say that “Unfortunately, Sr isotopic analysis of waterlogged wood has proven impossible” but then you state “This leads to the inclusion of archaeological timbers that cannot be dated and to innovative techniques being used to complement the picture such as aDNA analysis, elemental and isotopic analysis (O,N,C and Sr).” These are contradictory statements or I did not get your idea…

These are not contradictory waterlogged is the central problem here. It is waterlogging that produces a change in Sr isotopic signature, if the wood is not waterlogged, then there is no problem.

“In our case, since we are looking at forested areas that are still forested and with broadly the same species, this should not be a problem.” How do you know that the area was forested in the 16th century?

From historical records

I do not understand this sentence “However, it does mean that sampling oaks when studying oaks is to be preferred over sampling other species.” What else do you want sample if you study oaks? I am sorry but this sentence sounds crazy to me.

We are studying the Sr isotopic composition of timbers, this as explained in the paper reflects the bio-available Sr at the location of growth. One could use other proxies to determine the bio-availble Sr, maps created to study human mobility, Sr isotopic composition of bones from small rodents, different plant species. Other proxies are often used (read papers from Frei and Frei, Snoeck et al…). However, if at all possible the same species should be preferred since they get their nutrients from exactly the same depth and incorporate them in exactly the same way.

I do not agree with this sentence. “Charred vessels offer one very good example of wood material that cannot be provenanced through classical dendroarchaeological means due to the low number of rings.” Charring is not the cause of low number of rings. Charcoal samples can have enough rings for dating.

It's the fact that they are vessels that causes the low number of rings, not the charring, the sentence is indeed confusing. But in the new version this sentence is absent and the case-study only mentioned in a table.

“This brief survey of the literature…..” It is NOT brief. It seems to me that you write a review in some paragraphs.

It has now been shortened.

The main objective and hypothesis of your study should be stated in the last paragraph of the Introduction. You have hypothesis and goals spread out after the Introduction which makes it difficult for the readers to orientate in the text

This has been added.

I think that the chapter “Sr isotopic baselines in Scandinavia” should be a part of site description in the methods section.

No this should not be the case, this is not methods, but information from literature.

Materials and methods

Methods must be better described! Methodology of dendroprovenancing is missing. I think that numbering of chapters would be very helpful. Identification of samples should be clearly described in the methods. The section is very confusing. You work with recent and historical material but it is NOT clear at all.

We apologize for it not being clear, we have worked on rephrasing things to facilitate the understanding. We understand the frustration with the absence of numbering for sections, however that is how plosone works.

The aim of the study should not be part of the site description. Is the site Jylland or Jutland (in figure)?

Jylland is not a site, it is a region of Denmark called Jylland in Danish and Jutland in English. We have made this uniform.

How do you know that historical samples are from Denmark, Sweden and Norway? I think that such hypothesis should be tested on living trees where you exactly know the origin of the wood.

We expected them based on dendrochronological data to be from these regions (which is reflected in the sentence we believe, not we know). And we wanted to prove this using Sr isotopic analysis. Comparing the isotopic signature from the samples with values from literature for the different regions of interest.

Content of subchapters Tiendeladen, Brix Gard, Horsens are results of dendroprovenancing, aren´t they? It looks like; therefore, it belongs to results, not to methods!

This is a valid remark. Strictly speaking these are n0t results of this particular study, since they were reported on in separate dendrochronological studies and the sample selection for the Sr isotopic analysis was performed based on these results. However, since those reports are not published in the scientific literature, we restructured the paper to only keep the site description in the materials and methods, and put the dendrochronological part in the results.

Table 1 and 3 – What correlation? Pearson? Did you use t-test? Which one? Is the filename important? Why did you do not describe the last column?

The correlation is Baillie & Pilcher 1973. This information is now inserted in the table captions and references. The filename is the identifier in the authors’ database. The title of the last column inserted

In subchapter Horsens you mention F4 but it is not in Table 2.

Sample F4 was analysed dendrochronologically but never analysed for Sr. it is now identified in table 2. We plan to analyse it, but due to instrumental problems this has not yet been possible.

Suddenly, you say what aDNA analysis reveals but you did not describe how you performed the analysis.

This is part of a different publication by another group, so that does not need to be described and discussed here, the reference is also present.

Subchapter Horsens, 2nd paragraph – why do you think that t=6.25 is low?

Enough to date not enough to provenance. This is re-phrased in the manuscript.

Subchapter Horsens, 3rd paragraph – “it is likely that they are from trees of quite different provenances” Why do you think so?

To underline that point we have inserted, in that sentence, the phrase “given that they display low correlation with each other”

Subchapter Sr isotopes along the Gota-river – Suddenly you started to describe locations in Sweden but in the previous text you described results! I miss a description of the locations and a map. It is not easy to find orientation without a map.

Adapted. Map made also

I do not understand what you mean: “Water samples were simply dried down.” Did you dry water?

The water was evaporated. Dried down is the usual way of describing this in literature.

“these steps sometimes need to be repeated a number of times” Why? How many times?

That depends, they are repeated until complete dissolution. Usually dried leaves dissolve more easily than green wood. If the samples are somewhat thicker more cycles are also needed.

Results
Table 5 – It should be better described including identification of samples.

The identification of the samples is found in 4, but now we have repeated that information in table 5. We do not see how they can be better described. All the values are present in the table and are discussed in group.

Discussion

What are the expectations?

That there would be no difference (as seen in the next sentence).

These sentences are redundant “(1) since the leaves were lying on the floor the wood
and leaves could be from a different tree. This is a poor argument because it would mean that trees separated by only a few meters could have vastly different signatures.”

Done

“the leaves in Lödöse and Torskog had signatures almost identical to the soil values.” Do you mean Sr values or values of 87Sr/86Sr

Sr isotopic as this whole paragraph is on that subject. This is repeated in the sentence now.

“dead leaves exchanged Sr with the surrounding soil”- I guess you mean rather contamination than exchange.

No we mean exchange – contamination would yield increased Sr concentrations, and lead to an isotopic composition which would be a mixed signature between the tree and the soil. In this case the concentration is not increased and the isotopic composition is the same as in the soil. So presumably, as is the case in waterlogged context, the original Sr was completely replaced by exogeneous Sr.

Figure 2 is not well described and some letters are too small. What do the percentage and the symbols mean?

The percentages and the symbols are from the original publication of the figure– this is the legend: “Fig. 6. Locations of the 1187 analysed Ap and Gr sites with scaled symbols according to 87Sr/86Sr values percentiles and ordinary kriging interpolation in Surfer (Golden Software, 2017) on a 25 × 25 km grid, with 400 km search radius. The circles (values above median 0.7091) and squares (values below median) show the locations of the measured 50 km × 50 km sites and 87Sr/86Sr value for each grid cell is indicated on the colour legend.”

Rather than using this figure, we have used the figures produced from the R-script that goes with the paper to calculate probability of provenance.

|  | [Sr] (ng/g) | ^87^Sr/^86^Sr | 2sd |
| --- | --- | --- | --- |
| F2 | 3.14 | 0.715594 | 0.000127 |
| F3 | 1.91 | 0.712705 | 0.000008 |
| F6 | 4.15 | 0.712245 | 0.000045 |
| F7 | 25.21 | 0.708565 | 0.000032 |
| F8 | 3.40 | 0.710085 | 0.000076 |
| F9 | 2.20 | 0.709158 | 0.000037 |
| F10 | 2.55 | 0.709365 | 0.000012 |
| F11 | 2.90 | 0.709385 | 0.000037 |
| H1 | 8.17 | 0.716856 | 0.000023 |
| H2 | 2.83 | 0.715971 | 0.000076 |
| H3 | 0.93 | 0.716292 | 0.000049 |
| H4 | 6.56 | 0.722123 | 0.000039 |
| H5 | 2.45 | 0.716472 | 0.00006 |
| X2 | 1.089 | 0.712270 | 0.000004 |

“The cluster of samples from Tiendeladen indicates a provenance in South-Western Sweden, further south than Gothenburg.” Based on what can you say that? I guess that you cannot state so only based on the map in Fig. 2. It is not clear to me how you can exclude other parts of Scandinavia or Portugal and claim that samples are from south-western Sweden. It must be clarified and better explained in the manuscript. Maybe you can say based on the results of both methods…?

This figure has been replaced by figures actually showing the statistical modelling of the provenance and this whole section was rewritten.

“The single sample from Brix Gård has a signature that also matches Southern Sweden”. How does a signature of the samples match with signature of southern Sweden? Is it just because of similar values? Or any statistics? What is the probability?

Yes it is “just” because of the similar values. This is how isotopic data usually is used, unlike dendrochronological data. We did some modelling using the tool from Hoogewerf.

“Dendrochronology is a powerful tool for identifying the region of origin of historic timber. One of the potential biases in the method is the use of a dataset that in itself has a history of transport and re-use” I think that this idea should be better described in the manuscript. Do you think that you can develop a chronology from a mixture of local and imported timber? Do you think it is also possible in the case when you use living oaks from Denmark and historical constructions including oaks from e.g. southern Germany? Do you think that individual tree ring series from distances so far apart would be cross-dated well? Or do you mean biases at smaller geographical areas?

We have added an additional description here. We do not propose that large regional chronologies have been built from timber from such wide distances, but we use site chronologies extensively. This is exactly to avoid making large chronologies of timber of different origins.

Reviewer 2:

We thank the reviewer for its commendation of our work and for our efforts to speak to a wide audience. Since reviewer 1 was more skeptical about this, the whole text was reworked thoroughly, some of those explanations were removed, but we believe it still provides a good introduction to the subject matter.

Because a complementary method to dendroprovenancing is being tested here, this term should also appear in the keywords.

Thank you, this is done!

The years should be supplemented in the title ("Provenancing 16th and 17th century CE ...”) and in the continuous text by the information "CE" (formerly "AD"), so that the chronological
classification is unquestionable for readers of all disciplines. The religion-related "AD" (anno
domini) should also be replaced in Figure 1 by "CE", which is now common in the natural sciences.

Done, and done, thank you.

Chemical formulae should be resolved in subsequent brackets when first mentioned in the running
text, e.g. "NH3NO3 (ammonium nitrate)", or listed as a separate list/table at the end of the
article.

done

It is not clear whether the tree ring width curves of the wood samples are single measurements of one radius or mean curves from several radii. I think it is methodologically important to specify
this. If one follows the information in the S4-file (S4 file Van Ham-Meert & Daly 2022.fh), which
contains the data of the tree ring widths in the so-called Heidelberg format, it seems to be
exclusively a matter of single radii. If this is the case, I consider this methodologically
questionable as far as the dendrochronological dating of the timbers is concerned. It would also be useful if these tree-ring width data in the *.fh file contained information about the "location". Currently, besides the tree ring widths, they only contain information on dating, number of tree rings, species, pith, sapwood rings and keycode (see following example).

The dendrochronology methodology is not questionable, these measurements are checked extensively, and correlations at the dating positions are highly well replicated. Additionally, all measurements are made available, so peers can check these. Methodology is described now in more detail in the manuscript.

Done: location info inserted in tree-ring dataset file.

HEADER:
DateEnd=1556 Length=128 Species=QUSP Pith=1 SapWoodRings=2 KeyCode=H011001A DATA:Tree
149 113 113 99 81 77 67 77 79 53
76 59 69 69 82 49 63 44 70 80
60 73 70 54 103 86 94 75 73 68
83 67 61 56 59 52 53 46 66 72
58 43 45 45 44 63 71 72 56 56
38 57 33 53 67 49 43 49 68 65
49 45 32 29 47 55 52 65 60 54
57 57 78 63 84 92 159 117 146 144
139 143 130 155 168 135 175 151 144 131
89 63 60 120 82 76 133 150 130 113
95 133 112 138 136 88 113 126 115 98
106 115 112 121 114 105 133 123 133 155
104 96 112 80 188 234 262 217 0 0

In the text and in the illustrations, t-values are repeatedly mentioned to express the level of the
dendrochronological correlation of the timbers to each other. Unfortunately, nowhere is it
mentioned whether these values were calculated according to Student (1908), Baillie/Pilcher (1973) or Hollstein (1980). Also, values are described as significant or non-significant without
explaining which thresholds the authors use for this.

Done. threshold levels are given now in the text

Sometimes the information on the unit of measurement is added to the values without a space ("...500mg ..."), sometimes there is a space before it ("... 200 μL ..."). The spelling should be consistent according to the guidelines of PLOS ONE.

done

Under "Materials and Methods - Sites" it should be mentioned that the sapwood statistics are those for oaks, and that the Brix Gård site is not mentioned there because it is bark edge wood. For the Tiendeladen site it should be mentioned that the best correlating chronology for groups 1 and 2 comes from Gammel Strand C, because there are three chronologies from Gammel Strand (B, C and E) in Table 1. Here, too, significance is mentioned at the end of the section without discussing at which t-value the authors apply significance ("However, for both these groups the correlation with the few Western Swedish chronologies (i.e. with samples found inside Sweden) is less significant."). For non-dendrochronologists, it can also be confusing when, on the one hand, t-values above 10 are considered best dates in the text and, on the other hand, samples H4 and H5 are considered to be from the same stem (stem-matched) because the t-value is above 10.

Done (sapwood stats are mentioned in the fig 1 caption, but now inserted in the text as well). Other points (both long and short) added where appropriate

The heading of Table 1 is "Correlation between the three tree-ring groups in this study that dendrochronologically indicate a western Swedish provenance." But it is probably not the correlations between the three tree-ring groups, but the correlations of the individual groups to the "Master and site chronologies" and the "Chronologies from ships". This should be reworded.

Done

It should also be mentioned in all tables that the values given are t-values according to either Student (1908) or Baillie/Pilcher (1973) or Hollstein (1980). In the continuous text, one should consistently repeat the t-values from the tables in brackets and name the chronologies completely. This makes it easier to understand the argumentation, e.g. at the Brix Gård site, where it says „The dendrochronological correlations show highest agreement with material from Odense, Copenhagen [41] and Helsingør (table 1) but also with the group 3 timber from the Vasa ship [8] and a ship from Oslo (Barcode 14 [58]) both of which are probably from western Sweden. My suggestion would be: The dendrochronological correlations show highest agreement with material from Odense (TBT group 4, 9.78), Copenhagen (Gammel Strand B [41], 8.83) and Helsingør (8.46) (table 1) but also with the group 3 timber from the Vasa ship [8] (9.15) and a ship from Oslo
(Barcode 14 [58], 9.10) both of which are probably from western Sweden.
In Table 3 for the site Nørregade 12, Horsens, a chronology "Jutland/Funen" is mentioned, which
were named "Jylland or Fyn" in the continuous text above ("These five samples best match a
chronology for Jylland or Fyn (table 3)."). For a reader unfamiliar with the geographical
designations of Denmark, such inconsistencies can be irritating. Thus, all the designations in the
Supporting Information should be compared with those in the text.

done

The heading of Table 4 is "Sample locations and description in Sweden.”. Here I would change the heading of the fourth column "Sr" to "Sr-sample sources". In the accompanying text, it should be explained why the sampling of the four individual locations is inconsistent in type and extent (there are surely good reasons for that).

Indeed the last column heading was strange, this was adapted to sample type. Yes, we should explain the sampling strategy a little bit better, thank you.

The chemical formulae used in the section "Sr isotopic analysis - Sr leaching and sample digestions" I would, as already said in the introduction, either immediately dissolve in brackets, e.g. "HNO3 (nitric acid)", or dissolve all the formulae used in a table. In this section it also says "... these steps sometimes need to be repeated a number of times." Why is that?

The fresher the wood, the harder is it to dissolve, if the shavings are larger you also need more steps to completely dissolve it. Leaves were easier to dissolve than some of the wood.

In the section "Results - "Baseline data", the first paragraph reads "... (17x times more in the case of Torskog 2 and 4 times more in the case of Lödöse)." The "x" as a multiplication sign is probably a remnant of an earlier version of the text.

Well spotted thanks!

It is a little irritating that the values of the isotope ratios in Table 5 are given with 6 decimal places, but in the running text only 4 or 3 decimal places are mentioned. „The two samples from the Gota river (LEH201 and LEH2O2) have the highest Sr isotopic signature (a range of 0.7242-0.7246 is obtained when the measurement error is taken into account).” If we use Table 5 to calculate the measurement error (twofold standard deviation), we obtain more precise values (0.724187- 0.724625) and recognise that the authors have rounded up or down. This is probably due to better clarity.

Yes, this is what we did, we are unsure whether you would like this to be changed?

In Tables 5 and 6, the heading of column 4 should be named "± 2sd" instead of "2sd". This would make it clearer that a twofold standard deviation is meant here.

Done

In Table 5, the country name "Sweden" should be added to the heading, and in Table 6, "Denmark" should be added: „Table 5: Results from Sr isotopic determination of samples along the Gota river, Sweden.“, “Table 6: Srisotopic composition of timbers from 3 buildings in Denmark.”

done

In the section "Buildings", a number error has crept into the first line: „Four of the 5 samples from Tiendeladen cluster together 87Sr/86Sr = 0.175896-0.716879, only H4 has a slightly higher signature 0.722123 ± 0.000039 (table 6).” It must read “… 87Sr/86Sr= 0.715895-0.716879 …”.

Thank you

A small mistake has been made in the caption to Figure 2: „Figure 2. Summary of the results of the strontium isotope analysis for the timber from the three buildings in this study, placed together with the map reproduced from Hoogewerff [et.al](http://et.al/). [71], fig 6. (2 column figure)”. Should read “… from Hoogewerff et al. [71], …)

This figure has been removed and replaced

Figure subheadings or headings (figures, tables) should end consistently either with or without a full stop.

Proposals regarding content The claim made in the abstract "By adding the Sr isotopic analysis, a far more detailed interpretation of the origin of these timbers can be presented" cannot be confirmed in the paper. The authors themselves contradict this claim in their conclusion when they write, for example:"Dendrochronology provided more precise provenance for samples F7-F11 than Sr isotopes could.”

Sometime they provide more precise locations, sometimes they don’t, it depends on the signature. You are right, this is now adapted

The problem that reference curves/master chronologies were often constructed from timbers from different regions, and that for some historical reference curves it is not possible to know exactly from which regions the timbers originate, should be made clearer with 2-3 sentences (e.g. Ernst Hollstein and Bernd Becker have integrated sample material from each other's working area for certain weakly replicated periods).

done

The phenomenon of timber transport/trading has not only been a problem of dendrochronology and dendroprovenancing since the mid-14th century CE. In the chapter "Timber trade in Southern Scandinavia" it is correctly stated that "Gradually, from around the mid-14th century onwards, wood and timber in Northern Europe was traded from regions with more abundant forests to regions wherethese materials were in high demand and no longer locally available.”. Elsewhere ("Danish buildingswith varied timber sources") it then irritatingly states "From the mid-13th century onwards, oakwas shipped from the regions south and east of the Baltic Sea, in the form of boards of varyingsizes.".Apart from these incongruent chronological approaches (mid-13th vs. mid-14th century CE), it would also be desirable for less historically educated readers to point out that extensive timbertransport already took place in the Roman Empire (here Roman Imperial Period to Late Antiquity, c.27 BCE to mid-6th century CE). There is an extensive literature on this, most recently for exampleBernabei, M., Bontadi, J., Rea, R., Büntgen, U., Tegel, W. (2019): Dendrochronological evidence forlong-distance timber trading in the Roman Empire. PLoS ONE 14(12): e0224077.<https://journals.plos.org/plosone/article?id=10.1371/journal.pone.0224077>

done

One could also briefly mention that supra-regional timber transport in prehistoric times in Europeup to the Roman period is not a problem for provenance research on timbers: the place where thetimbers processed by humans were found largely corresponds to the place where the trees grew.This changes temporarily in Roman times, but to my knowledge has not yet been documented for thesubsequent early and high Middle Ages (mid-6th century to mid-13th century CE). From the Late Middle Ages onwards (mid-13th century CE), the transport of timber in Europe resumed.

done

The sentence following Table 3, "Using extensive tree-ring datasets across the region, and examining the highest correlations geographically, the region where the timber achieves highest correlation can be pinpointed as the region where the tree grew.", I would add to the content that ecologically similar growth conditions are possible in different regions and can lead to similar growth curves (tree-ring width data). This is a critical point in dendrochronological provenance determination (dendroprovenancing), and it should at least be mentioned, because it cannot be ruled out that comparative data are not available from all regions and that one therefore only recognizes similarities with the areas already researched, which do not necessarily have to be the region of origin.

Have written a detailed summary of the technique, including the phrase: “…but it is also important to look at the geographical distribution of these correlations, and to be aware of what regions might be lacking relevant tree-ring datasets, when making conclusions”

The sentence „Hence, we want to use strontium isotopic analysis to be able to pinpoint the source of the timber, not just where it was used, but where the trees grew.” does not seem logical to me because the SIA is not supposed to clarify the place of use.

Indeed, this is re-written

In the section "Sr isotopes along the Gota-river" the colleagues mentioned should also be named:"This data completes earlier datasets collected by colleagues". Which colleagues are these?

This sentence is no longer present, but in the section on baselines the colleague sare appropriately cited.

The problem of (undesirable) contamination of oak leaves by rainwater leaching should already be addressed in the following sentence and not further down in the text: „Oak tree leaves were brown and collected at the foot of oak trees in forested areas.” In the section "Results - Baseline data" that follows below, it then states quite correctly “The dead leaves probably had leached some of their Sr and some Sr was replaced by less radiogenic Sr contained in rainwater.”

In the section “Combining the analysis techniques - …” it says “One of the potential biases in the method is the use of a dataset that in itself has a history of transport and re-use. This necessitates careful interpretation of dendrochronological provenance results in order to avoid circular arguments.” Here it would be appropriate to also refer in the text to the need for the construction of "clean" regional and also supra-regional chronologies in which the origin of all wood samples is known. This is, after all, precisely the problem discussed in the essay, which the SIA is supposed to help with.

done

In the following sentence „Using strontium isotopic analysis to interrogate key questions of the dendrochronological dataset an extra level of accuracy is achieved”, I would phrase it more neutrally like this: “Using strontium isotopic analysis to interrogate key questions of the dendrochronological dataset an extra level of information is achieved”. The accuracy is not really that much better, as the authors themselves write in the following "Conclusion". Rather, added value is achieved through the additional safeguarding with an independent proxy, which, however, is only possible for dry woods. (see passages underlined below): „It showed the added value of Sr isotopic analysis on dry building timbers as illustrated in the case study of Brix Gård. Dendrochronology provided more precise provenance for samples F7-F11 than Sr isotopes could. Whereas the analysis of sample F3 prompted a re-examination of the dendrochronological data to a Southern Swedish rather than Danish provenance. For the pine samples the dendrochronology identified them as originating from two locations in Norway. Sr isotopic analysis confirmed that the two pines had different provenances”. I would make the last part of the sentence, in bold, more neutral: „Sr isotopic analysis revealed that pines had different Sr-signatures supporting the dendrochronological results of different provenances.”

Thank you for this remark, this was adapted
