# Supplementary material for: Provenancing 16th and 17th century CE building timbers in Denmark–combining dendroprovenance and Sr isotopic analysis
Source: PLoS One. 2023 Feb 9;18(2):e0278513. doi: 10.1371/journal.pone.0278513 (PMC9910641; doi:10.1371/journal.pone.0278513)
Supplement: Attachment — Submitted filename: Aoife replies review 2.docx [file pone.0278513.s007.docx]

We thank the reviewer for his kind words and his help in improving the manuscript. We also value his openness on leaving some statements he might not entirely agree with and trusting our scientific work.

I still disagree with you that measuring a single radius of tree-ring widths is sufficient. It may be sufficient for a secure dating in the cases presented here, and I trust completely that the authors have secured their dating very well. But for basic statistical considerations, at least a second control measurement should always be made for tree-ring width measurements. This may sound "oldschool", but it is important and remains correct. I do not expect any changes in your text in this respect, but I would be pleased if you would take my advice to heart and implement it in the future as a contrast to a "one radius quick & dirty" dendrochronology.

Thank you for that. AD has indeed taken your advice wholly to heart, and is much more frequently measuring multiple radii to strengthen the dataset.

In your conclusion you wrote:
"For the pine samples the dendrochronology identified them as originating from two locations in Norway. Sr isotopic analysis confirmed that the two pines had different provenances."
And I suggested that the second sentence be worded more neutrally:
"Sr isotopic analysis revealed that pines had different Sr-signatures supporting the dendrochronological results of different provenances."
It is a pity that you could not come to terms with this. But leave it as it is. It is your statement.

We are happy to change this sentence to a more neutral wording. Thank you for your suggestion

However, I have two things to note that I insist on changing:

1. In the file "S4 file Van Ham-Meert & Daly 2022.fh" the location information is still missing, although the reply to the reviewer says: "Done: location info inserted in tree-ring dataset file." Have I perhaps received an old version of the file here?

You are right it seems we did not upload an updated version, this should now be the case.

2. In table 7, column 4, the "±" sign was forgotten: "±2sd" instead of "2sd". Please correct.

Of course, thank you for seeing this!
